# Primary internal Iliac Aneurysm-Rectal Fistula treated with a combined endovascular and endoscopic approach as a palliative strategy: a case report

**DOI:** 10.3389/fcvm.2025.1658009

**Published:** 2025-10-21

**Authors:** Tao Zhang, Dongxingyu Li, Xiyang Chen, Hankui Hu

**Affiliations:** ^1^ Division of Outpatient, West China Hospital of Sichuan University, Chengdu, China; ^2^ Day Surgery Center, West China TianFu Hospital, Sichuan University, Chengdu, Sichuan, China; ^3^ Division of Vascular Surgery, Department of General Surgery, West China Hospital of Sichuan University, Chengdu, Sichuan, China

**Keywords:** arterio-intestine fistula, endoscopy, endovascular repair, internal iliac aneurysm, digestive endoscopic hemostasis

## Abstract

**Background:**

A primary aortoenteric fistula (AEF) is a pathologic communication between the aorta and the gastrointestinal tract. Although uncommon, this condition carries a substantial risk of life-threatening hemorrhage.

**Case presentation:**

An 82-year-old male with a history of coronary artery disease and chronic obstructive pulmonary disease (COPD) presented to the emergency department with acute gastrointestinal bleeding. Computed tomography (CT) revealed bilateral common iliac artery aneurysms and left internal iliac artery aneurysm. In addition, the rectal wall demonstrated heterogeneous thickening with an apparent focal discontinuity along the proximal left lateral wall, suggestive of a fistulous tract between the left internal iliac artery and the rectum. Emergency intervention was performed, which included embolization of the inflow and outflow tracts of the left internal iliac artery aneurysm using fibered coils, followed by deployment of a covered stent from the origin of the left common iliac artery to the left external iliac artery. The patient experienced recurrent gastrointestinal bleeding postoperatively, which was managed with endoscopic hemostasis. He was discharged on postoperative day 15 after the initial procedure and remained well during a 7-month follow-up period.

**Conclusion:**

Early and accurate diagnosis of aortoenteric fistula is paramount. Endovascular repair combined with endoscopic hemostasis can serve as an effective bridging or palliative strategy to stabilize patients and create a window for definitive surgery, despite this patient demonstrated a favorable short-term outcome.

## Introduction

Primary AEF is a relatively uncommon yet highly lethal condition. Historically, delays in diagnosis and management have been associated with exceedingly high mortality rates due to its rapid and fulminant course (1–3). Arterio-gastrointestinal fistulas may occur in patients with thoraco-abdominal aortic aneurysm, abdominal aortic aneurysm, common iliac artery aneurysm and splenic artery aneurysm (4–6). Following erosion into the gastrointestinal tract, the aneurysm may rupture into the intestinal lumen, resulting in massive hemorrhage. This manifests as acute gastrointestinal bleeding, which can be misdiagnosed as benign gastrointestinal hemorrhage, leading to critical delays in diagnosis and treatment and potentially catastrophic outcomes (3). Therefore, timely and accurate diagnosis and intervention are critical to preventing catastrophic outcomes. Currently, many reports of AEF involving the duodenum exist (4), but only a few cases of internal iliac aneurysm-rectal or sigmoid fistula have been reported (2, 3, 7–10), and the treatment options include open surgical repair, such as reconstruction *in situ* or anatomic external bypass, and endovascular repair (5). The endovascular treatment of aneurysm-enteric fistulas still faces numerous challenges, such as persistent gastrointestinal bleeding/ulcers after endovascular therapy, graft infection, hemorrhage during aneurysm surgery, and postoperative infection risks. The purpose of this case is to report the successful treatment of an internal iliac aneurysm-rectal fistula through combined endovascular and endoscopic intervention. This case report has been written in line with the SCARE criteria (11).

## Case presentation

**Figure 1:**
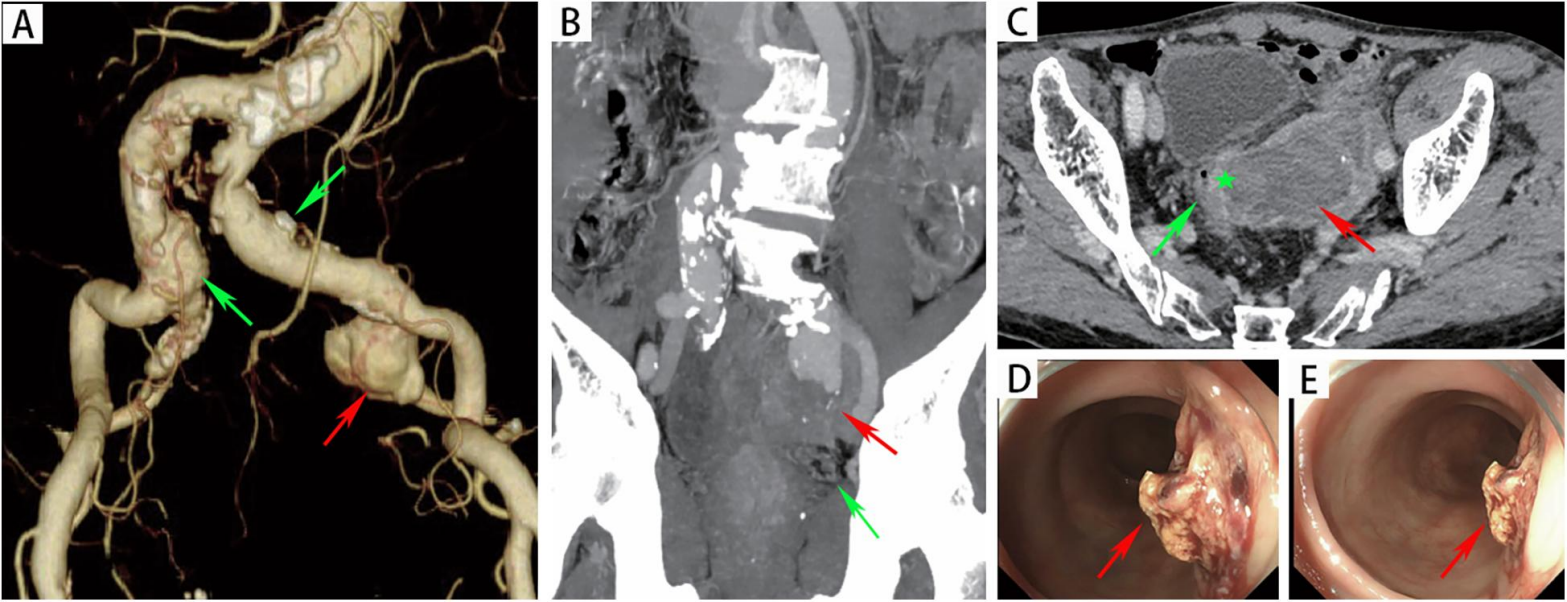
**(A)** CTA revealed a left internal iliac aneurysm (red arrow) and bilateral common iliac artery aneurysms (green arrow). **(B)** The red arrow indicated the left internal iliac artery aneurysm, with the rectum (green arrow) located below it. **(C)** CTA revealed that there was an unclear boundary between the left internal iliac artery aneurysm (red arrow) and the rectal wall (green pentagon). **(D-E)** Endoscopic evaluation revealed a raised, blood clot-adherent ulcer (arrows) on the rectal lateral wall.

An 82-year-old male presented to the emergency department with a half-month history of intermittent hematochezia, with an estimated blood loss of 100–200 ml per episode. He also reported generalized weakness, dizziness, lower abdominal pain, and occasional passage of blood clots. The patient denied abdominal distension, diarrhea, fever, or bowel obstruction, and reported no significant recent weight change. His medical history included coronary artery disease, smoking, and COPD. There was no prior surgical or trauma history. Physical examination revealed a pulsatile mass in the left lower abdomen without significant tenderness. The extremities were cool and clammy, with poor peripheral perfusion. Vital signs on admission were: temperature 36.3°C, heart rate 122 beats/min, respiratory rate 23 breaths/min and blood pressure 115/78 mmHg. Laboratory investigations revealed anemia (hemoglobin:75 g/L; reference range: 114–154 g/L), hypoalbuminemia (albumin:25.8 g/d; reference range: 40–55 g/L), renal insufficiency (serum creatinine:1.34 mg/dl; reference range: 0.6–1.2 mg/dl) and acute heart failure (B-type natriuretic peptide: 1,125 ng/L; reference range < 226 ng/L). Other parameters were within normal limits. Computed tomographic angiography (CTA) demonstrated a 32 × 27 mm right common iliac artery aneurysm and a 20 × 27 mm left common iliac artery aneurysm. A 66 × 51 mm aneurysm was identified in the left internal iliac artery with extensive intramural thrombus (Figure 1A). Furthermore, the rectal wall showed irregular thickening with focal discontinuity along the left upper wall (Figures 1B,C), suggestive of a fistula between the left internal iliac artery aneurysm and the rectum. Emergency colonoscopy revealed an ulcer with active bleeding at 13 cm from the anal verge (Figures 1D,E); a biopsy of the surrounding tissue was obtained. The patient was diagnosed with: (1) left internal iliac artery–rectal fistula; (2) hemorrhagic shock; (3) hypoalbuminemia; (4) renal insufficiency; (5) coronary artery disease; (6) acute heart failure and (7) COPD.

**Figure 2:**
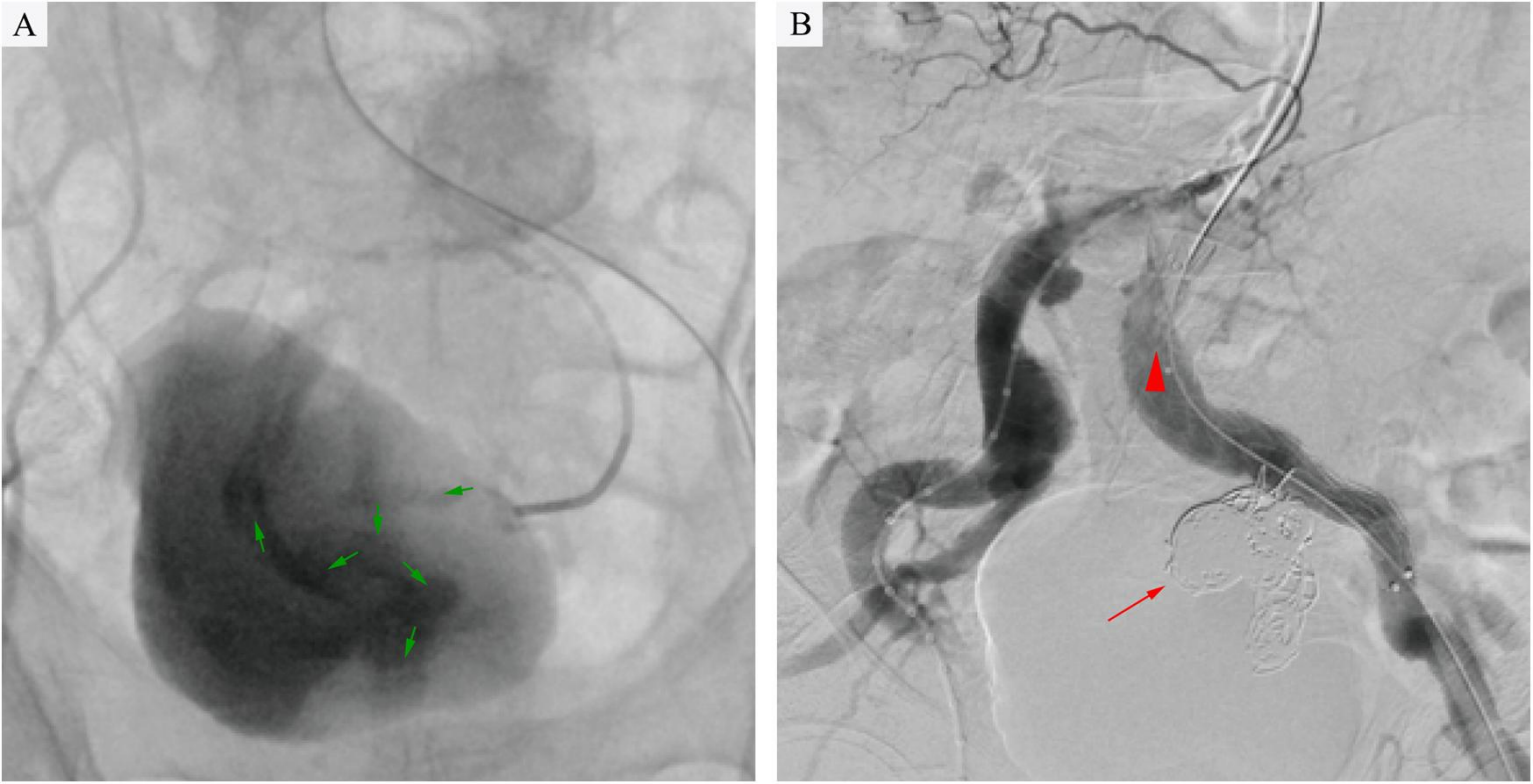
**(A)** Intraoperative angiography revealed contrast extravasation (green arrows) into the rectal lumen, confirming an internal iliac artery aneurysm-rectal fistula following selective catheterization of the left internal iliac artery aneurysm. **(B)** Angiography confirmed complete aneurysm exclusion after coil embolization (red arrow) and stent-graft deployment (red triangle), with no residual contrast leakage into the rectum.

A multidisciplinary team comprising specialists in vascular surgery, anesthesiology, gastrointestinal surgery, and gastroenterology, in conjunction with the patient's family's preferences, concluded that endovascular intervention by vascular surgery was necessary given the patient's hemorrhagic shock and severe comorbidities. The procedure was performed as follows: after local infiltration of lidocaine to both groins, the common femoral arteries were accessed bilaterally. Two Perclose ProGlide™ devices (Abbott Vascular, CA, USA) were pre-deployed in the left common femoral artery, followed by insertion of a 12F sheath (Cook Medical). Through a 5F sheath on the right, a pigtail catheter was advanced to the infrarenal aorta, where angiography demonstrated bilateral common iliac artery aneurysms and a left internal iliac artery aneurysm. Using a crossover technique, a catheter was positioned in the left common iliac artery to obtain roadmap guidance. It was then selectively advanced into the aneurysm sac. Hand-injected angiography revealed the outflow vessel and contrast agent leakage into the rectal colon (Figure 2A). A microcatheter and microwire (Cook Medical) were used to select the outflow vessel, and fibered coils (MicroNester® Embolization Coils: 4mm × 2, 3mm × 5, 2mm × 5) were deployed for complete embolization. The catheter was withdrawn into the aneurysm sac, where additional coils (MReye® Embolization Coils:20mm × 6, 18mm × 4, 12mm × 5,10mm × 3, 8mm × 3) were placed to achieve dense packing. The catheter was further withdrawn to the inflow zone of the aneurysm, where embolization was completed using fibered coils (MicroNester® Embolization Coils: 4mm × 3, 3mm × 4, 2mm × 5). Post-embolization hand-injected contrast via the left sheath showed no opacification of the left internal iliac artery, with preserved flow in the external iliac artery. A covered stent graft (Endurant II 16 × 10 × 156 mm, Medtronic) was deployed from the origin of the left common iliac artery to the mid portion of the external iliac artery. Completion angiography confirmed patency of the left external iliac artery and complete occlusion of the left internal iliac artery (Figure 2B). Postoperative management included parenteral nutrition, blood transfusion, and antibiotic therapy (levofloxacin).

**Figure 3:**
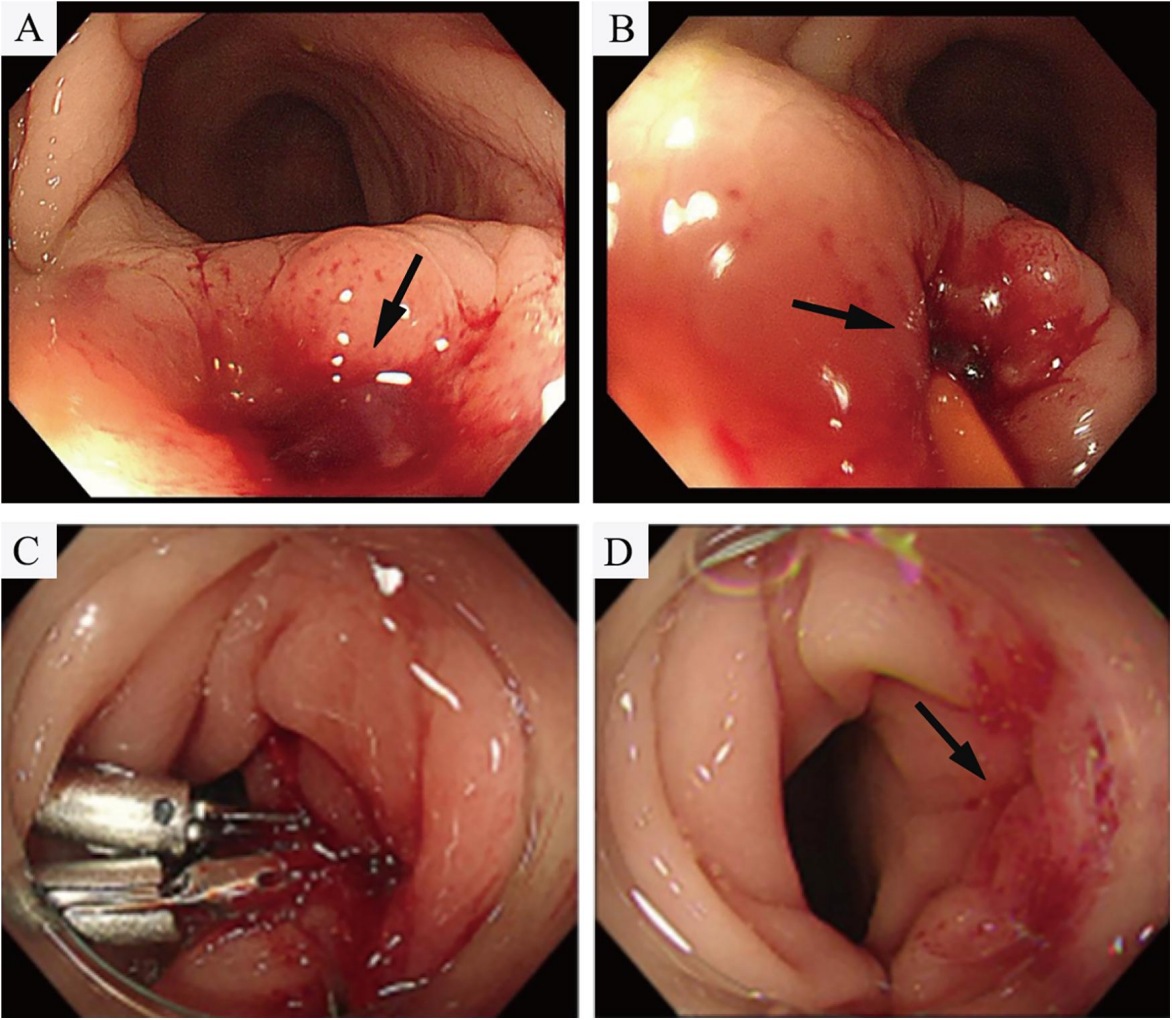
**(A,B)** Colonoscopy demonstrated tissue necrosis and fibrinous exudate at the fistula site, with minimal oozing (arrowheads). **(C)** Hemostasis was achieved via through-the-scope endoscopic clips. **(D)** On day 11 after endoscopic hemostasis, follow-up endoscopy demonstrated revealed complete fistula closure with mild residual mucosal edema.

**Figure 4:**
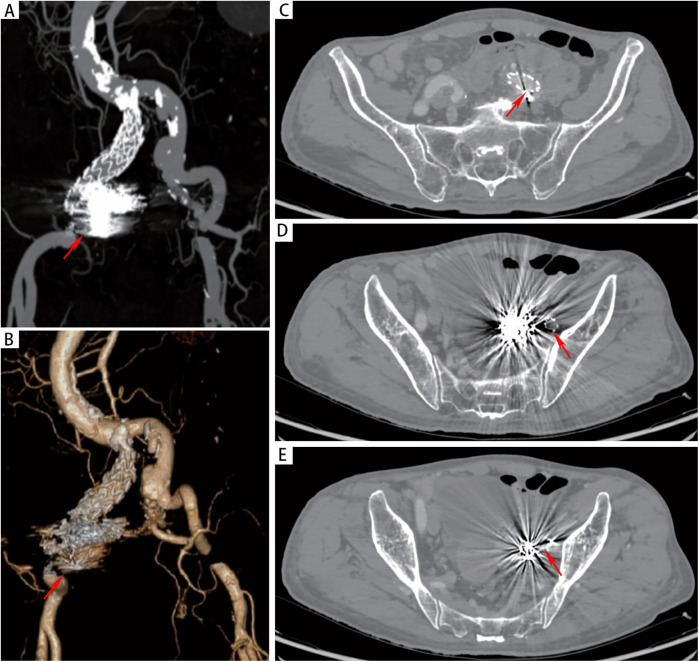
**(A,B)** CTA at 7 months postoperatively demonstrated patency of the left iliac artery stent and complete occlusion of the left internal iliac artery. **(C-E)** CT demonstrated absence of gas accumulation in the perigraft region.

**Figure 5:**
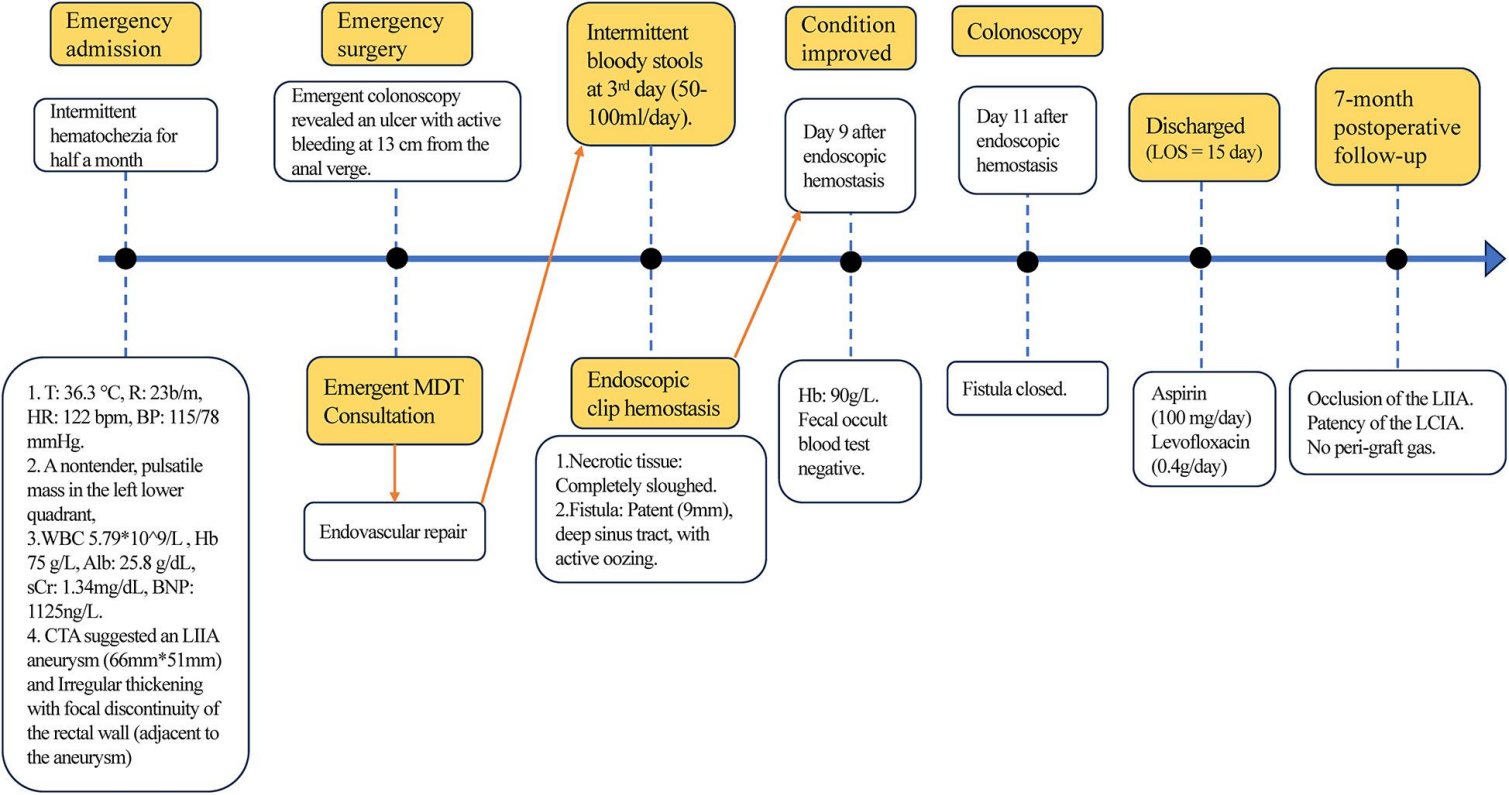
The timeline of therapeutic interventions. T: Temperature. MDT: Multidisciplinary Team Consultation.

However, on the third day after the endovascular procedure, the patient experienced intermittent lower gastrointestinal bleeding (50–100 ml/day). Colonoscopy was subsequently performed, which revealed complete sloughing of the necrotic tissue at the enteric fistula site, exposing a fistula tract approximately 9 mm in diameter with active oozing (Figures 3A,B). Endoscopic clip hemostasis was then performed (Figure 3C). Following endoscopic hemostasis, the patient was maintained on parenteral nutrition and antibiotics, with close monitoring of hemoglobin levels and fecal occult blood tests. During this period, the patient remained afebrile without chills or fever. Three sets of blood cultures were negative. Hemoglobin levels gradually increased, and the frequency and volume of bloody stools steadily decreased. On post-endoscopic hemostasis day 9, the hemoglobin level was 90 g/L and the fecal occult blood test was negative. On day 11 after endoscopic hemostasis, follow-up endoscopy demonstrated complete closure of the fistula with only mild residual mucosal edema (Figure 3D). Biopsy specimens obtained during the initial emergency endoscopy showed chronic mucosal inflammation, focal necrosis with cholesterol deposition, mixed acute and chronic inflammatory cell infiltration, and multinucleated giant cell reaction. The patient resumed oral intake on day 12 after the initial surgery and was discharged on day 15 without complications. The discharge regimen included enteric-coated aspirin (100 mg/day) and levofloxacin (0.4 g/day). During a 7-month telephone follow-up, the patient reported normal bowel movements without fever or abdominal pain. A follow-up CT scan performed 7 months postoperatively demonstrated complete occlusion of the left internal iliac artery (Figure 4A), patency of the left external iliac artery (Figure 4B), and the stent without foci of gas (Figures 4C–E). The detailed sequence of therapeutic interventions is presented in the treatment timeline (Figure 5). Informed consent was obtained from both the patients and their families.

## Discussion

AEF is a rare condition in which an aneurysm compresses the intestinal tract, causing necrosis of both the intestinal and aneurysm walls, ultimately leading to fistula formation. Aneurysms are often asymptomatic initially, and even after AEF develops, symptoms may be subtle or entirely absent. Nevertheless, AEF is life-threatening. Prompt, accurate diagnosis and management are critical for patients with aneurysms suggesting impending or active rupture associated with an enteric fistula. However, misdiagnosis as gastrointestinal bleeding frequently leads to inappropriate treatment and delayed intervention (3, 12). Physical examination may disclose a tender, pulsatile abdominal mass. CTA demonstrating close contact between an aneurysm and bowel should raise strong suspicion for AEF. Characteristic findings include periaortic gas, loss of the arterial–enteric fat plane, intraluminal contrast extravasation, and peri-aortic bowel wall edema (2).

Management of AEF aims to control hemorrhage, prevent infection, and maintain distal perfusion. Surgical options include endovascular aneurysm repair (EVAR) and open surgical repair (OSR). However, OSR is associated with limited survival benefit and poor postoperative quality of life in many patients, often due to advanced age, comorbidities, and surgical site infection (4, 13).Aortic reconstruction may involve extra-anatomic bypass with aortic ligation or *in situ* grafting, frequently supplemented with rifampicin soaking and omental coverage (4, 14). Colonic fistulae entail higher infection risk than duodenal fistulae. For aorto-duodenal fistulas, extra-anatomic bypass (e.g., femoro-femoral crossover) offers a less complex alternative. Although extra-anatomic bypass avoids heavily contaminated areas, persistent bacteremia may still cause graft infection. Subsequent graft excision due to infection results in significantly greater tissue damage than initial surgery.

Endovascular repair serves as an emergent, bridging, or even palliative intervention for AEF, aimed at stabilizing patients by addressing hemorrhagic shock, systemic infection, and underlying comorbidities, thereby optimizing conditions for eventual open surgical repair. The primary concern with endovascular repair as a first-line strategy is graft infection. To date, 6 studies (2, 3, 7–10) have reported endovascular management of iliac artery–colonic fistulae. Tatsuishi et al. (7) successfully treated an AEF using a stent graft (Gore Excluder AAA Endoprosthesis system, USA), while Bhatti et al. (8) employed a Viabahn (Gore, USA) stent graft for a left external iliac artery aneurysm–enteric fistula. Although both cases involved bloodstream infection, prolonged antibiotic therapy resulted in favorable short-term outcomes. Khalaf et al. (9) reported stent infection following endovascular repair, necessitating subsequent open surgical reconstruction. Four studies (3, 8–10) described endoscopic findings: two identified a pulsating mass on the bowel wall, one procedure was aborted due to active bleeding, and one found no definitive fistula. Noda et al. (14) reported successful endovascular combined with endoscopic clip hemostasis for a primary aortoduodenal fistula in a 91-year-old patient, with good outcomes at 6-month follow-up. Similarly, we report a case of iliac artery aneurysm–enteric fistula successfully managed via combined endovascular and endoscopic intervention, with a favorable outcome observed during the 7-month follow-up. Although no evidence of graft infection was detected during follow-up, it remains a significant concern. Open surgery remains the preferred option for surgical candidates, while endovascular repair may serve as a bridge or palliative therapy in patients with life-threatening bleeding and severe comorbidities or poor functional status to facilitate eventual open repair. No formal guidelines currently exist regarding the duration of antibiotic therapy after endovascular repair for AEF; however, we recommend long-term antibiotic treatment. Discontinuation should be guided by endoscopic ulcer healing and consistently negative blood cultures for over six months. This study has several limitations: the follow-up period was relatively short, and although no signs of graft infection have been observed thus far, extended surveillance is necessary to assess long-term graft integrity. Additionally, due to the emergent nature of the procedure, certain angiographic sequences were not optimally captured to fully illustrate the procedural details.

## Conclusion

Endovascular repair represents a treatment option for patients with iliac artery aneurysms associated with enteric fistula. Endoscopic intervention may be considered in cases with persistent gastrointestinal bleeding following the procedure, although vigilant monitoring for graft infection is essential. It must be emphasized that open surgical repair remains the most reliable therapeutic approach.

## Data Availability

The original contributions presented in the study are included in the article/Supplementary Material, further inquiries can be directed to the corresponding author/s.
